# Free energy asymptotics of the quantum Heisenberg spin chain

**DOI:** 10.1007/s11005-021-01375-4

**Published:** 2021-03-09

**Authors:** Marcin Napiórkowski, Robert Seiringer

**Affiliations:** 1 Department of Mathematical Methods in Physics, Faculty of Physics, University of Warsaw, Pasteura 5, 02-093 Warsaw Poland; 2 Institute of Science and Technology Austria, Am Campus 1, 3400 Klosterneuburg, Austria

**Keywords:** Quantum spin chains, Heisenberg model, Ferromagnet, Free energy, Spin waves, Bose gas, 82B10, 82D40, 81Q10

## Abstract

We consider the ferromagnetic quantum Heisenberg model in one dimension, for any spin $S\ge 1/2$. We give upper and lower bounds on the free energy, proving that at low temperature it is asymptotically equal to the one of an ideal Bose gas of magnons, as predicted by the spin-wave approximation. The trial state used in the upper bound yields an analogous estimate also in the case of two spatial dimensions, which is believed to be sharp at low temperature.

## Introduction

The ferromagnetic quantum Heisenberg model is one of the most important and widely studied models of statistical mechanics. In dimensions $d \ge 3$, the model is widely believed to display long-range order at low temperature, but a rigorous proof remains elusive. Based on the concept of long-range order, the low temperature properties of the model are usually examined using spin-wave theory. In the spin-wave approximation, one assumes that the low-energy behavior of the system can be described in terms of collective excitations of spins called spin waves. From an equivalent point of view, which dates back to Holstein and Primakoff [17], these spin waves are known as bosonic quasiparticles called magnons.

The spin-wave approximation has been very successful, predicting for example a phase transition in three and more dimensions, or the $T^{3/2}$ Bloch magnetization law [7, 8]. In his seminal 1956 paper [14], Dyson derived further properties of the quantum Heisenberg model which, among other things, included the low temperature expansion of the magnetization.

While there was little doubt about the validity of spin-wave theory in three (or more) dimensions, a rigorous proof of some of its predictions has only recently been given in [13] (see also [12]). There it was proved that the free energy of the three-dimensional ferromagnetic quantum Heisenberg model is, to leading order, indeed given by the expression derived using spin-wave approximation, for any spin $S\ge 1/2$ (see also [10, 25] for earlier non-sharp upper bounds, or [5, 11] for results in the large *S* limit).

The situation is different in lower dimensions. It has been known since the seminal work of Mermin and Wagner [19] that the $d=1$ and $d=2$ dimensional quantum Heisenberg models do not exhibit long-range order at any nonzero temperature. The low temperature behavior of the system in low dimensions is thus very different from the one in three or higher dimensions, and it is less clear whether spin-wave theory should also be valid in lower dimensions.

In 1971, Takahashi [22] derived a free energy expansion for $d=1$ in the case $S=1/2$. In this special case, the quantum Heisenberg model is exactly solvable via the Bethe ansatz [6]. The spectrum of the (finite size) model can be obtained by solving the corresponding Bethe equations. Under certain assumptions (known as string hypothesis) on the solutions of these equations, he derived what are now known as thermodynamic Bethe equations, an analysis of which leads to a formula for the free energy. Later, in [23] he derived an alternative free energy expansion using (a modified) spin-wave theory (for any *S*, and also in two dimensions). Interestingly, the second terms in the (low temperature) free energy expansions in [22, 23] do not agree with the predictions of conventional spin-wave theory [7, 8, 14, 17]. (The leading terms do agree, however.)

The thermodynamic Bethe equations have been used not only for the Heisenberg spin chain, but also in other models including the Kondo model [1–3, 21] or the Gross–Neveu model in high energy physics [4]. For more applications of the string hypothesis and its relation to numerous other models in physics, we refer to the review articles [18, 24].

In the present paper, using different methods, we prove that, to leading order, the formula derived by Takahashi based on the Bethe ansatz and the string hypothesis in [22] is indeed correct. Our analysis does not use the Bethe ansatz and our result holds for any spin *S*. It therefore also partly justifies the spin-wave approximation derived in [23]. We shall utilize some of the methods developed for the three-dimensional case in [13], but novel ingredients are needed to treat the case of lower dimensions, both for the upper and the lower bounds.

## Model and main result

We consider the one-dimensional ferromagnetic quantum Heisenberg model with nearest neighbor interactions. For a chain of length *L*, it is defined in terms of the Hamiltonian

2.1 $$\begin{aligned} H_L = \sum _{x=1}^{L-1} \left( S^2 - \vec {S}_x \cdot \vec {S}_{x+1} \right) . \end{aligned}$$

Here, ${\vec {S}}=(S^1,S^2,S^3)$ denote the three components of the spin operators corresponding to spin *S*, i.e., they are the generators of the rotations in a $2S+1$-dimensional representation of *SU*(2). The Hamiltonian $H_L$ acts on the Hilbert space ${\mathscr {H}}_L = \bigotimes _{x=1}^L {\mathbb {C}}^{2S+1}$. We added a constant $S^2$ for every bond in order to normalize the ground state energy of $H_L$ to zero.

Our main object of study is the specific free energy

$$\begin{aligned} f_L(\beta ,S) = - \frac{1}{\beta L} \ln \left( {{\,\mathrm{Tr}\,}}e^{-\beta H_L} \right) \end{aligned}$$

for $\beta >0$, and its thermodynamic limit

2.2 $$\begin{aligned} f(\beta ,S) = \lim _{L\rightarrow \infty } f_{L}(\beta ,S). \end{aligned}$$

We are interested in the behavior of $f(S,\beta )$ in the low temperature limit $\beta \rightarrow \infty$ for fixed *S*. Our main result is as follows.

### Theorem 2.1

Consider the Hamiltonian (2.1) and the corresponding free energy (2.2). For any $S\ge 1/2$,

2.3 $$\begin{aligned} \lim _{\beta \rightarrow \infty } f(\beta ,S)S^{\frac{1}{2}} \beta ^{\frac{3}{2}}=C_1:=\frac{1}{2\pi }\int _{{\mathbb {R}}}\ln \big (1-e^{-p^2}\big )\mathrm{d}p=\frac{-\zeta (\frac{3}{2})}{2\sqrt{\pi }}, \end{aligned}$$

where $\zeta$ denotes the Riemann zeta function.

The proof of Theorem 2.1 will be given in Sects. 4 and 5, where we derive quantitative upper and lower bounds, respectively. The trial state employed in the derivation of the upper bound can also be used in $d=2$ dimensions. We refer to Proposition A.1 in Appendix A for a precise statement and its proof. A corresponding lower bound for $d=2$ is still missing, however.

The analogue of Theorem 2.1 for $d=3$ was proved in [13]. While the new tools developed here for the lower bound use the one-dimensional nature of the model in an essential way, they are robust enough to allow for an extension of our results to quasi-one-dimensional systems, like Heisenberg models defined on ladder graphs. Such an extension is rather straightforward and we shall not give the details here.

## Boson representation

It is well known that the Heisenberg Hamiltonian can be rewritten in terms of bosonic creation and annihilation operators [17]. For any $x \in [1,\ldots , L] \subset {\mathbb {Z}}$, we set

3.1 $$\begin{aligned} S_{x}^+ = \sqrt{2S}\, a^\dagger _x \left[ 1 - \frac{a^\dagger _x a_x}{2S} \right] _+^{1/2} \ , \, S_{x}^{-} = \sqrt{2S}\left[ 1 - \frac{a^\dagger _x a_x}{2S} \right] _+^{1/2} a_x\ , \,\, S_{x}^3 = a^\dagger _x a_x - S\,,\nonumber \\ \end{aligned}$$

where $a^{\dagger }_{x}, a_x$ are bosonic creation and annihilation operators, $S^\pm _x = S^1_x \pm i S^2_x$, and $[\, \cdot \,]_+ = \max \{0, \, \cdot \, \}$ denotes the positive part. The operators $a^\dagger$ and *a* act on $f \in \ell ^2({\mathbb {N}}_0)$ via $(a\, f)(n) = \sqrt{n+1} f(n+1)$ and $(a^\dagger f)(n) = \sqrt{n} f(n-1)$, and satisfy the canonical commutation relations $[a,a^\dagger ] = 1$. One readily checks that (3.1) defines a representation of *SU*(2) of spin *S*, and the operators ${\vec {S}}_x$ leave the space $\bigotimes _{x=1}^L \ell ^2 ( [0,2S]) \cong {\mathscr {H}}_L = \bigotimes _{x=1}^L {\mathbb {C}}^{2S+1}$, which can naturally be identified with a subspace of the Fock space ${\mathcal {F}}_L:=\bigotimes _{x=1}^L \ell ^2({\mathbb {N}}_0)$, invariant.

The Hamiltonian $H_L$ in (2.1) can be expressed in terms of the bosonic creation and annihilation operators as

3.2 $$\begin{aligned} H_L&= S \sum _{x=1}^{L-1} \biggl ( - a^\dagger _x \sqrt{ 1 - \frac{n_x}{2S} } \sqrt{ 1-\frac{n_{x+1}}{2S}}a_{x+1} - a^\dagger _{x+1} \sqrt{ 1-\frac{n_{x+1}}{2S}} \sqrt{ 1 - \frac{n_x}{2S} } a_x \nonumber \\&\qquad \qquad + n_x + n_{x+1} - \frac{1}{S} n_x n_{x+1} \biggl ) \,, \end{aligned}$$

where we denote the number of particles at site *x* by $n_x= a^\dagger _x a_x$. It describes a system of bosons hopping on the chain $[1,\ldots L]$ with nearest neighbor *attractive* interactions and a hard-core condition preventing more than 2*S* particles to occupy the same site. Also the hopping amplitude depends on the number of particles on neighboring sites, via the square root factors in the first line in (3.2).

In the bosonic representation (3.2), the Fock space vacuum $|\Omega \rangle$ (defined by $a_x |\Omega \rangle = 0$ for all *x*) is a ground state of the Hamiltonian $H_L$, and the excitations of the model can be described as bosonic particles in the same way as phonons in crystals. There exists a zero-energy ground state for any particle number less or equal to 2*SL*, in fact. While this may not be immediately apparent from the representation (3.2), it is a result of the *SU*(2) symmetry of the model. The total spin is maximal in the ground state, which is therefore $(2SL+1)$-fold degenerate, corresponding to the different values of the 3-component of the total spin. The latter, in turn, corresponds to the total particle number (minus *SL*) in the bosonic language.

Before we present the proof of Theorem 2.1, we shall briefly explain the additional difficulties compared to the $d=3$ case, and the reason why the proof in [13] does not extend to $d=1$. Spin-wave theory predicts that at low temperatures the interaction between spin waves can be neglected to leading order. This means that (3.2) can effectively be replaced by the Hamiltonian of free bosons hopping on the lattice. At low temperature and long wavelengths $\ell \gg 1$, one can work in a continuum approximation where the last term $-\sum _x n_x n_{x+1}$ in (3.2) scales as $\ell ^{-d}$, while the kinetic energy scales as $\ell ^{-2}$. The interaction terms can thus be expected to be negligible only for $d\ge 3$, and this is indeed what was proved in [13]. This argument is in fact misleading, as the attractive interaction term turns out to be compensated by the correction terms in the kinetic energy coming from the square root factors. Making use of this cancellation will be crucial for our analysis (while it was not needed in [13] to derive the free energy asymptotics for $d\ge 3$).

We note that for $d=1$ and $d=2$ the interaction is strong enough to create bound states between magnons [15, 16, 20, 26, 27]. These occur only at nonzero total momentum, however, with binding energy much smaller than the center-of-mass kinetic energy at low energies. Hence, they do not influence the thermodynamic properties of the system at low temperature to leading order.

## Upper bound

Recall the definition of $C_1$ in (2.3). In this section, we will prove the following.

### Proposition 4.1

As $\beta S \rightarrow \infty$, we have

4.1 $$\begin{aligned} f(\beta ,S) \le C_1 S^{-\frac{1}{2}} \beta ^{-\frac{3}{2}} \left( 1 - {\mathcal {O}}( (\beta S)^{-\frac{1}{8}} (\ln \beta S)^{3/4}) \right) .\, \end{aligned}$$

The general structure of the proof will be similar to the corresponding upper bound given in [13]. The difference lies in the choice of the trial state, which in contrast to [13] allows for more than one particle on a single site. This is essential in order to capture the desired cancellations explained in the previous section.

*Step 1. Localization in Dirichlet boxes.* Our proof will rely on the Gibbs variational principle, which states that

4.2 $$\begin{aligned} f_L (\beta ,S) \le \frac{1}{L} {{\,\mathrm{Tr}\,}}H_L \Gamma + \frac{1}{\beta L} {{\,\mathrm{Tr}\,}}\Gamma \ln \Gamma \end{aligned}$$

for any positive $\Gamma$ with ${{\,\mathrm{Tr}\,}}\Gamma =1$. We shall confine the particles into smaller intervals, introducing Dirichlet boundary conditions. To be precise, let

$$\begin{aligned} H_{L}^\mathrm{D} = H_{L} + 2 S^2 + S(S_1^3 + S_L^3) \end{aligned}$$

be the Heisenberg Hamiltonian on $\Lambda _L := [1,\ldots ,L] \subset {\mathbb {Z}}$ with $S^3_x=-S$ boundary conditions. Note that $H_{L}^\mathrm{D} \ge H_{L}$. It is well known that the thermodynamic limit in (2.2) exists, hence we can assume without loss of generality that $L = k(\ell +1)+1$ for some integers *k* and $\ell$. By letting all spins on the boundary of the smaller intervals of side length $\ell$ point maximally in the negative 3-direction, we obtain the upper bound

$$\begin{aligned} f_{L}(\beta ,S) \le \left( 1 + \ell ^{-1} \right) ^{-1} f_{\ell }^\mathrm{D}(\beta ,S) \ , \quad f_\ell ^\mathrm{D}(\beta ,S) := - \frac{1}{\beta \ell } \ln \left( {{\,\mathrm{Tr}\,}}e^{-\beta H_{\ell }^\mathrm{D}} \right) \,. \end{aligned}$$

In particular, by letting $k\rightarrow \infty$ for fixed $\ell$, we have

4.3 $$\begin{aligned} f(\beta ,S) \le \left( 1 + \ell ^{-1} \right) ^{-1} f_{\ell }^\mathrm{D}(\beta ,S) \end{aligned}$$

in the thermodynamic limit.

*Step 2. Choice of trial state.* To obtain an upper bound on $f_{\ell }^\mathrm{D}$, we can use the variational principle (4.2), with

4.4 $$\begin{aligned} \Gamma = \frac{ {\mathcal {P}} e^{-\beta K} {\mathcal {P}} }{{{\,\mathrm{Tr}\,}}_{\mathcal {F}}{\mathcal {P}} e^{-\beta K }{\mathcal {P}}} \, \end{aligned}$$

where we denote the Fock space ${\mathcal {F}}\equiv {\mathcal {F}}_\ell$ for simplicity. Here, ${\mathcal {P}}$ is an operator satisfying $0\le {\mathcal {P}}\le 1$, and is defined by

4.5 $$\begin{aligned} {\mathcal {P}}=\prod _{x=1}^{\ell }f(n_x) \end{aligned}$$

where

4.6 $$\begin{aligned} f(n)= {\left\{ \begin{array}{ll} 1 &{} \text {if} \quad n=0; \\ \left[ \prod _{j=1}^{n}\left( 1-\frac{j-1}{2S}\right) \right] ^{\frac{1}{2}} &{} \text {if} \quad n=1,2,\ldots , 2S;\\ 0 &{} \text {if} \quad n>2S. \end{array}\right. } \end{aligned}$$

Note that $0\le {\mathcal {P}}\le 1$, and ${\mathcal {P}}$ is zero if more than 2*S* particles occupy some site. The operator *K* is the Hamiltonian on Fock space ${\mathcal {F}}$ describing free bosons on $\Lambda _{\ell }=[1,\ldots ,\ell ]$ with Dirichlet boundary conditions, i.e.,

4.7 $$\begin{aligned} K&= S \sum _{x,y \in \Lambda _\ell } \left( -\Delta ^\mathrm{D}\right) (x,y) a^\dagger _x a_y \nonumber \\&= S \sum _{\langle x,y\rangle \subset \Lambda _\ell } \left( - a^\dagger _x a_y - a^\dagger _y a_x + n_x + n_y \right) + S (n_1 + n_\ell ) \end{aligned}$$

where $\Delta ^\mathrm{D}$ denotes the Dirichlet Laplacian on $\Lambda _\ell$ and $\langle x,y\rangle$ means that *x* and *y* are nearest neighbors. The eigenvalues of $-\Delta ^\mathrm{D}$ are given by

4.8 $$\begin{aligned} \left\{ \varepsilon (p) = 2 (1-\cos (p)) \, : \, p \in \Lambda _\ell ^{*\mathrm D}:= \left\{ \frac{k\pi }{\ell +1}: k\in \{ 1,\ldots , \ell \} \right\} \right\} \end{aligned}$$

with corresponding eigenfunctions $\phi _p(x) = [2/(\ell +1)]^{\frac{1}{2}} \sin (x p)$.

*Step 3. Energy estimate.* We shall now give a bound on the energy of the trial state.

### Lemma 4.1

On the Fock space ${\mathcal {F}}=\bigotimes _{x\in \Lambda _\ell } \ell ^2({\mathbb {N}}_0)$,

4.9 $$\begin{aligned} {\mathcal {P}} H_{\ell }^\mathrm{D} {\mathcal {P}} \le K\,. \end{aligned}$$

### Proof

Definition (4.5) implies that

4.10 $$\begin{aligned} \begin{aligned} {\mathcal {P}}a^\dagger _x= & {} \prod _{z\in \Lambda _\ell }f(n_z) a^\dagger _x=a^\dagger _x f(n_x+1)\prod _{\underset{z\ne x}{z\in \Lambda _\ell }}f(n_z)=a_x^\dagger {\mathcal {P}}\sqrt{1-\frac{n_x}{2S}}. \end{aligned} \end{aligned}$$

It follows that

4.11 $$\begin{aligned} {\mathcal {P}}a^\dagger _x \sqrt{ 1 - \frac{n_x}{2S} } \sqrt{ 1-\frac{n_y}{2S}}a_y {\mathcal {P}}= a^\dagger _x {\mathcal {P}}^2 \bigg (1-\frac{n_x}{2S}\bigg )\bigg (1-\frac{n_y}{2S}\bigg ) a_y. \end{aligned}$$

With the aid of (4.10) and (4.11), one checks that

$$\begin{aligned} \begin{aligned} {\mathcal {P}} H_{\ell }^\mathrm{D} {\mathcal {P}}&= S\sum _{\langle x,y\rangle \subset \Lambda _\ell } (a^\dagger _x - a^\dagger _y) {\mathcal {P}}^2 \bigg (1-\frac{n_x}{2S}\bigg )\bigg (1-\frac{n_y}{2S}\bigg )(a_x-a_y) \\&\quad + S \sum _{x\in \{1,\ell \}} a^\dagger _x {\mathcal {P}}^2 \bigg (1-\frac{n_x}{2S}\bigg ) a_x. \end{aligned} \end{aligned}$$

The desired bound (4.9) then follows directly from ${\mathcal {P}}^2 \big (1-\frac{n_x}{2S}\big )\big (1-\frac{n_y}{2S}\big )\le 1$ and ${\mathcal {P}}^2 \big (1-\frac{n_x}{2S}\big )\le 1$. $\square$

We conclude that

4.12 $$\begin{aligned} {{\,\mathrm{Tr}\,}}H_{\ell }^\mathrm{D} \Gamma \le \frac{{{\,\mathrm{Tr}\,}}_{\mathcal {F}}K e^{-\beta K}}{{{\,\mathrm{Tr}\,}}_{\mathcal {F}}{\mathcal {P}}e^{-\beta K}{\mathcal {P}}}\, . \end{aligned}$$

As a next step, we will show that ${{\,\mathrm{Tr}\,}}_{\mathcal {F}}{\mathcal {P}} e^{-\beta K}{\mathcal {P}}$ is close to ${{\,\mathrm{Tr}\,}}_{\mathcal {F}}e^{-\beta K}$ for $\ell \ll (\beta S)^\frac{2}{3}$. The following lemma is an adaptation of the corresponding result in [13, Lemma 4.3].

### Lemma 4.2

We have

4.13 $$\begin{aligned} \frac{ {{\,\mathrm{Tr}\,}}_{\mathcal {F}}{\mathcal {P}} e^{-\beta K}{\mathcal {P}}}{{{\,\mathrm{Tr}\,}}_{\mathcal {F}}e^{-\beta K} } \ge 1- \left( \frac{\pi ^2}{12}\right) ^2 \frac{\ell (\ell +1)^2}{(\beta S)^2}. \end{aligned}$$

### Proof

Using that $f(n_x)\le 1$ and that $f(n_x)=1$ if $n_x\in \{0,1\}$, we have

4.14 $$\begin{aligned} 1-{\mathcal {P}}^2 \le \sum _{x=1}^\ell (1-f^2(n_x)) \le \frac{1}{2}\sum _{x=1}^\ell n_x (n_x-1) = \frac{1}{2} \sum _{x=1}^\ell a^\dagger _x a^\dagger _x a_x a_x \,. \end{aligned}$$

Wick’s rule for Gaussian states therefore implies that

4.15 $$\begin{aligned} \frac{ {{\,\mathrm{Tr}\,}}_{\mathcal {F}}{\mathcal {P}} e^{-\beta K}{\mathcal {P}}}{{{\,\mathrm{Tr}\,}}_{\mathcal {F}}e^{-\beta K} } \ge 1- \frac{1}{2} \sum _{x=1}^\ell \frac{ {{\,\mathrm{Tr}\,}}_{\mathcal {F}}a^\dagger _x a^\dagger _x a_x a_x e^{-\beta K}}{{{\,\mathrm{Tr}\,}}_{\mathcal {F}}e^{-\beta K} } = 1 -\sum _{x=1}^\ell \left( \frac{ {{\,\mathrm{Tr}\,}}_{\mathcal {F}}n_x e^{-\beta K}}{{{\,\mathrm{Tr}\,}}_{\mathcal {F}}e^{-\beta K} }\right) ^2 \,.\nonumber \\ \end{aligned}$$

Moreover,

$$\begin{aligned} \frac{ {{\,\mathrm{Tr}\,}}_{\mathcal {F}}n_x e^{-\beta K}}{{{\,\mathrm{Tr}\,}}_{\mathcal {F}}e^{-\beta K}}= & {} \frac{1}{ e^{\beta S (-\Delta ^\mathrm{D})} -1 }(x,x) =\sum _{p \in \Lambda _\ell ^{*\mathrm D}}\frac{|\phi _p(x)|^2}{e^{\beta S \varepsilon (p)}-1}\\\le & {} \frac{2}{\ell +1}\sum _{p \in \Lambda _\ell ^{*\mathrm D}}\frac{1}{e^{\beta S \varepsilon (p)}-1}. \end{aligned}$$

By using $(e^{x}-1)^{-1}\le x^{-1}$ for $x\ge 0$ in the last sum, as well as $1-\cos x\ge \frac{2x^2}{\pi ^2}$ for $x\in (0,\pi )$, this gives

4.16 $$\begin{aligned} \frac{ {{\,\mathrm{Tr}\,}}_{\mathcal {F}}n_x e^{-\beta K}}{{{\,\mathrm{Tr}\,}}_{\mathcal {F}}e^{-\beta K}} \le \frac{\ell +1}{2 \beta S } \sum _{n=1}^\ell \frac{1}{n^2} \le \frac{\pi ^2}{12} \frac{\ell +1}{\beta S } \,. \end{aligned}$$

Inserting this bound into (4.15) yields the desired result. $\square$

*Step 4. Entropy estimate.* It remains to give a lower bound on $-{{\,\mathrm{Tr}\,}}\Gamma \ln \Gamma$, the entropy of $\Gamma$. We proceed in the same way as in [13, Lemma 4.4].

### Lemma 4.3

We have

$$\begin{aligned} \frac{1}{\beta }{{\,\mathrm{Tr}\,}}\Gamma \ln \Gamma&\le - \frac{1}{\beta }\ln \left( {{\,\mathrm{Tr}\,}}_{\mathcal {F}}{\mathcal {P}} e^{-\beta K}{\mathcal {P}}\right) - \frac{{{\,\mathrm{Tr}\,}}_{\mathcal {F}}K e^{-\beta K} }{{{\,\mathrm{Tr}\,}}_{\mathcal {F}}{\mathcal {P}} e^{-\beta K}{\mathcal {P}}} \\&\quad + S \left( \frac{\pi ^2}{12} \right) ^2 \frac{ \ell (\ell +1)^3}{(\beta S)^{7/2}} \left[ \frac{\sqrt{\pi }\zeta (3/2)}{8} + \frac{ (\beta S)^{1/2}}{\ell } \right] \frac{{{\,\mathrm{Tr}\,}}_{\mathcal {F}}e^{-\beta K} }{{{\,\mathrm{Tr}\,}}_{\mathcal {F}}{\mathcal {P}} e^{-\beta K}{\mathcal {P}}} \,. \end{aligned}$$

### Proof

We have

$$\begin{aligned} {{\,\mathrm{Tr}\,}}\Gamma \ln \Gamma = - \ln \left( {{\,\mathrm{Tr}\,}}_{\mathcal {F}}{\mathcal {P}} e^{-\beta K}{\mathcal {P}}\right) + \frac{1}{{{\,\mathrm{Tr}\,}}_{\mathcal {F}}{\mathcal {P}} e^{-\beta K} {\mathcal {P}}} {{\,\mathrm{Tr}\,}}_{\mathcal {F}}{\mathcal {P}} e^{-\beta K} {\mathcal {P}} \ln \left( {\mathcal {P}} e^{-\beta K} {\mathcal {P}} \right) \,. \end{aligned}$$

Using the operator monotonicity of the logarithm, as well as the fact that the spectra of ${\mathcal {P}} e^{-\beta K} {\mathcal {P}}$ and $e^{-\beta K/2} {\mathcal {P}}^2 e^{-\beta K/2}$ agree, we can bound

$$\begin{aligned} {{\,\mathrm{Tr}\,}}_{\mathcal {F}}&{\mathcal {P}} e^{-\beta K} {\mathcal {P}} \ln \left( {\mathcal {P}} e^{-\beta K} {\mathcal {P}} \right) = {{\,\mathrm{Tr}\,}}_{\mathcal {F}}e^{-\beta K/2} {\mathcal {P}}^2 e^{-\beta K/2} \ln \left( e^{-\beta K/2} {\mathcal {P}}^2 e^{-\beta K/2} \right) \\&\le {{\,\mathrm{Tr}\,}}_{\mathcal {F}}e^{-\beta K/2} {\mathcal {P}}^2 e^{-\beta K/2} \ln e^{-\beta K} = - \beta {{\,\mathrm{Tr}\,}}_{\mathcal {F}}K {\mathcal {P}}^2 e^{-\beta K} \,. \end{aligned}$$

Hence,

4.17 $$\begin{aligned} {{\,\mathrm{Tr}\,}}\Gamma \ln \Gamma \le - \ln \left( {{\,\mathrm{Tr}\,}}_{\mathcal {F}}{\mathcal {P}} e^{-\beta K} {\mathcal {P}}\right) - \beta \frac{{{\,\mathrm{Tr}\,}}_{\mathcal {F}}K e^{-\beta K} }{{{\,\mathrm{Tr}\,}}_{\mathcal {F}}{\mathcal {P}} e^{-\beta K} {\mathcal {P}}} + \beta \frac{{{\,\mathrm{Tr}\,}}_{\mathcal {F}}K (1-{\mathcal {P}}^2) e^{-\beta K} }{{{\,\mathrm{Tr}\,}}_{\mathcal {F}}{\mathcal {P}} e^{-\beta K}{\mathcal {P}}} \,.\nonumber \\ \end{aligned}$$

In the last term, we can bound $1-{\mathcal {P}}^2$ as in (4.14), and evaluate the resulting expression using Wick’s rule. With $\phi _p$ the eigenfunctions of the Dirichlet Laplacian, displayed below Eq. (4.8), we obtain

4.18 $$\begin{aligned} \begin{aligned} \frac{ {{\,\mathrm{Tr}\,}}_{\mathcal {F}}K n_x(n_x-1) e^{-\beta K} }{{{\,\mathrm{Tr}\,}}_{\mathcal {F}}e^{-\beta K} }&= \left( \frac{ {{\,\mathrm{Tr}\,}}_{\mathcal {F}}n_x e^{-\beta K} }{{{\,\mathrm{Tr}\,}}_{\mathcal {F}}e^{-\beta K} } \right) ^2 \sum _{p \in \Lambda _\ell ^{*\mathrm D}} \frac{2S \varepsilon (p) }{e^{\beta S \varepsilon (p)} -1} \\&\quad + \frac{ {{\,\mathrm{Tr}\,}}_{\mathcal {F}}n_x e^{-\beta K} }{{{\,\mathrm{Tr}\,}}_{\mathcal {F}}e^{-\beta K} } \sum _{p \in \Lambda _\ell ^{*\mathrm D}} \frac{ S\varepsilon (p) |\phi _p(x)|^2 }{ \left( \sinh \tfrac{1}{2} \beta S \varepsilon (p) \right) ^2 } \,. \end{aligned} \end{aligned}$$

The expectation value of $n_x$ can be bounded independently of *x* as in (4.16). When summing over *x*, we can use the normalization $\sum _x |\phi _p(x)|^2 =1$. To estimate the sums over *p*, we proceed similarly as in the proof of Lemma 4.2 to obtain

$$\begin{aligned} \sum _{p \in \Lambda _\ell ^{*\mathrm D}} \frac{2S \varepsilon (p) }{e^{\beta S \varepsilon (p)} -1}&\le \frac{\ell +1}{\pi } \int _{0}^\pi \frac{2S \varepsilon (p) }{e^{\beta S \varepsilon (p)} -1} \mathrm{d}p \le \frac{\ell +1}{\pi ^3} \int _{0}^\pi \frac{8 S p^2}{e^{4\beta S p^2/\pi ^2 } -1} \mathrm{d}p \\&\le S \frac{\ell +1}{(\beta S)^{3/2}} \int _0^\infty \frac{p^2}{e^{p^2} - 1} \mathrm{d}p = S \frac{\ell +1}{(\beta S)^{3/2}} \frac{\sqrt{\pi }}{4} \zeta (3/2) \end{aligned}$$

and

$$\begin{aligned} \sum _{p \in \Lambda _\ell ^{*\mathrm D}} \frac{ S\varepsilon (p) }{ \left( \sinh \tfrac{1}{2} \beta S \varepsilon (p) \right) ^2 } \le \frac{4}{S\beta ^2 } \sum _{p \in \Lambda _\ell ^{*\mathrm D}} \frac{ 1 }{ \varepsilon (p) } \le \frac{(\ell +1)^2}{S\beta ^2 } \sum _{n=1}^\ell \frac{ 1 }{ n^2 } \le \frac{\pi ^2}{6} \frac{(\ell +1)^2}{S\beta ^2} \,. \end{aligned}$$

In combination, this yields the desired bound. $\square$

*Step 5. Final estimate.* The Gibbs variational principle (4.2) together with (4.12) and Lemmas 4.3 and 4.2 implies that

$$\begin{aligned} \begin{aligned} f_\ell ^\mathrm{D}(\beta ,S)&\le - \frac{1}{\beta \ell } \ln \left( {{\,\mathrm{Tr}\,}}_{\mathcal {F}}{\mathcal {P}} e^{-\beta K}{\mathcal {P}}\right) + C S\frac{\ell ^3}{(\beta S)^{7/2}}\frac{{{\,\mathrm{Tr}\,}}_{\mathcal {F}}e^{-\beta K} }{{{\,\mathrm{Tr}\,}}_{\mathcal {F}}{\mathcal {P}} e^{-\beta K}{\mathcal {P}}}\\&\le -\frac{1}{\beta \ell }\ln \left( {{\,\mathrm{Tr}\,}}_{\mathcal {F}}e^{-\beta K} \right) -\frac{1}{\beta \ell }\ln \left( 1-\frac{C \ell ^3}{(\beta S)^2}\right) + C S\frac{\ell ^3}{(\beta S)^{7/2}} \end{aligned} \end{aligned}$$

for a suitable constant $C>0$, as long as $C (\beta S)^{1/2} \le \ell \ll (\beta S)^{2/3}$. The first term on the right side in the second line of the expression above equals

4.19 $$\begin{aligned} - \frac{1}{\beta \ell } \ln \left( {{\,\mathrm{Tr}\,}}_{\mathcal {F}}e^{-\beta K}\right) = \frac{1}{\beta \ell } \sum _{p\in \Lambda _\ell ^{*\mathrm D} } \ln ( 1- e^{-\beta S \varepsilon (p)} )\,. \end{aligned}$$

By monotonicity, we can bound the sum by the corresponding integral,

4.20 $$\begin{aligned} \frac{1}{\beta \ell } \sum _{p\in \Lambda _\ell ^{*\mathrm D} } \ln ( 1- e^{-\beta S \varepsilon (p)} ) \le \frac{1}{\pi \beta } \left( 1 +\ell ^{-1} \right) \int _{\frac{\pi }{\ell +1}}^{\pi } \ln ( 1- e^{-\beta S \varepsilon (p)} ) \mathrm{d}p\,, \end{aligned}$$

which is of the desired form, except for the missing part

$$\begin{aligned} - \frac{1}{\pi \beta } \int _0^{\frac{\pi }{\ell +1}} \ln ( 1- e^{-\beta S \varepsilon (p)} ) \mathrm{d}p\le & {} - \frac{1}{\beta (\ell +1)} \int _0^1 \ln \left( 1 - e^{- \frac{4 \beta S}{(\ell +1)^2} p^2} \right) \mathrm{d}p \\= & {} {\mathcal {O}}\left( \frac{ \ln (\ell ^2/(\beta S)) }{ \beta \ell }\right) \end{aligned}$$

for $\ell \gg (\beta S)^{1/2}$. Since $\varepsilon (p)\le p^2$, we further have

$$\begin{aligned} \frac{1}{\beta \pi } \int _{0}^\pi \ln ( 1- e^{-\beta S \varepsilon (p)} ) \mathrm{d}p&\le \frac{1}{\pi \beta } \int _{0}^\infty \ln ( 1- e^{-\beta S p^2} ) \mathrm{d}p + \frac{C}{\beta (\beta S)^\alpha } \\&=C_1 S^{-1/2} \beta ^{-3/2} + \frac{C}{\beta (\beta S)^\alpha } \end{aligned}$$

for arbitrary $\alpha >0$, some $C>0$ (depending on $\alpha$), and $C_1$ defined in (2.3). For $(\beta S)^{2/3} \gg \ell \gg (\beta S)^{1/2}$, all the error terms are small compared to the main term. The desired upper bound stated in Proposition 4.1 is obtained by combining the estimate above with (4.3) and choosing $\ell = C (\beta S)^{5/8} (\ln \beta S)^{1/4}$. $\square$

## Lower bound

Recall the definition (2.3) of $C_1$. In this section, we shall prove the following.

### Proposition 5.1

As $\beta S \rightarrow \infty$, we have

$$\begin{aligned} f(\beta ,S) \ge C_1 S^{-\frac{1}{2}} \beta ^{-\frac{3}{2}} \left( 1 + {\mathcal {O}}( (\beta S)^{-\frac{1}{12}}(\ln \beta S)^{1/2} (\ln \beta S^3)^{\frac{1}{3}}) \right) . \end{aligned}$$

Note that in contrast to the upper bound in Proposition 4.1, the lower bound above is not entirely uniform in *S*. Indeed, one has $\ln (\beta S^3)= \ln (\beta S)+\ln S^2$ and hence *S* is not allowed to grow arbitrarily fast compared to $\beta S$. To obtain a uniform bound, one can combine our results with the method in [11] where the case $S\rightarrow \infty$ for fixed $\beta S$ was analyzed.

The remainder of this section is devoted to the proof of Proposition 5.1. For clarity, the presentation will be divided into several steps. Some of them will use results from [13].

*Step 1. Localization.* Recall the definition (2.1) of the Hamiltonian $H_L$. For a lower bound, we can drop a term $( S^2 - \vec {S}_\ell \cdot \vec {S}_{\ell +1})$ from the Hamiltonian, which leads to the subadditivity

5.1 $$\begin{aligned} L f_L(\beta , S) \ge \ell f_{\ell }(\beta , S) + (L-\ell ) f_{L-\ell }(\beta ,S) \end{aligned}$$

for $1\le \ell \le L-1$. By applying this repeatedly, one readily finds that

$$\begin{aligned} f(\beta ,S) \ge f_\ell (\beta ,S) \end{aligned}$$

for any $\ell \ge 1$. We shall choose $\ell$ large compared with the thermal wavelength, i.e., $\ell \gg (\beta S)^{1/2}$.

*Step 2. Lower bound on the Hamiltonian.* Recall that the total spin operator is defined as $\vec {S}_\mathrm{tot} = \sum _{x=1}^\ell \vec {S}_x$. It follows from the theory of addition of angular momenta that

5.2 $$\begin{aligned} \vec {S}_\mathrm{tot}^2 = T(T+1) \ \text {with }\ \sigma (T)=\{0,1,\ldots , S\ell \}\,, \end{aligned}$$

where $\sigma$ denotes the spectrum. We will use the following bound on the Hamiltonian.

### Lemma 5.1

With *T* defined in (5.2), we have

5.3 $$\begin{aligned} H_\ell \ge \frac{2}{\ell ^3} ( S\ell (S \ell +1) - \vec {S}_\mathrm{tot}^2 ) \ge \frac{2S}{\ell ^2}\left( S\ell - T\right) . \end{aligned}$$

### Proof

It was shown in [13, Eq. (5.6)] that

$$\begin{aligned} (S^2-\vec {S}_x\cdot \vec {S}_y) + (S^2-\vec {S}_y\cdot \vec {S}_z)\ge \frac{1}{2} (S^2-\vec {S}_x\cdot \vec {S}_z) \end{aligned}$$

for three distinct sites *x*, *y*, *z*, and consequently that

$$\begin{aligned} (y-x) \sum _{w=x}^{y-1} \left( S^2 - \vec {S}_w \cdot \vec {S}_{w+1} \right) \ge \frac{1}{2} (S^2-\vec {S}_x\cdot \vec {S}_{y}) \end{aligned}$$

for any $x<y$. After summing the above bound over all $1\le x < y \le \ell$, we obtain

$$\begin{aligned} \sum _{1\le x<y\le \ell } (S^2-\vec {S}_x\cdot \vec {S}_{y})&\le 2 \sum _{1\le x<y\le \ell } (y-x) \sum _{w=x}^{y-1} \left( S^2 - \vec {S}_w \cdot \vec {S}_{w+1} \right) \\&= 2 \sum _{w=1}^{\ell -1} \left( S^2 - \vec {S}_w \cdot \vec {S}_{w+1} \right) \sum _{x=1}^w \sum _{y=w+1}^\ell (y-x). \end{aligned}$$

We have

$$\begin{aligned} \sum _{x=1}^w \sum _{y=w+1}^\ell (y-x) = \frac{\ell }{2} w (\ell - w) \le \frac{\ell ^3}{8} \end{aligned}$$

for $1\le w \le \ell -1$, and hence

$$\begin{aligned} H_\ell \ge \frac{4}{\ell ^3} \sum _{1\le x<y\le \ell } (S^2-\vec {S}_x\cdot \vec {S}_{y}) =\frac{2}{\ell ^3} ( S\ell (S \ell +1) - \vec {S}_\mathrm{tot}^2 ). \end{aligned}$$

As $\vec {S}_\mathrm{tot}^2 = T(T+1)$ we thus have

$$\begin{aligned} H_\ell \ge \frac{2S}{\ell ^2}\left( S\ell +1 - \frac{T(T+1)}{S\ell }\right) . \end{aligned}$$

The final bound (5.3) then follows from the fact that $T\le S\ell$. $\square$

Note that Lemma 5.3 implies, in particular, a lower bound of $2S \ell ^{-2}$ on the spectral gap of $H_\ell$ above its ground state energy. For $S=1/2$, it follows from the work in [9] that the exact spectral gap equals $( 1- \cos (\pi /\ell ))$ (which is $\frac{1}{2} \pi ^2 \ell ^{-2}$ to leading order for large $\ell$).

*Step 3. Preliminary lower bound on free energy.* With the aid of (5.3), we shall now prove the following preliminary lower bound on the free energy.

### Lemma 5.2

Let

5.4 $$\begin{aligned} \ell _0 := \sqrt{\frac{4 \beta S}{\ln \beta S }} \end{aligned}$$

and assume that $\ell \ge \ell _0/2$. Then, for $\beta S$ sufficiently large, we have

5.5 $$\begin{aligned} f_\ell (\beta ,S) \ge - C\frac{ \left( \ln \beta S\right) ^{1/2}}{\beta ^{3/2} S^{1/2}} \ln \beta S^3 \end{aligned}$$

for some constant $C>0$.

### Proof

With the aid of (5.3) and the *SU*(2) symmetry, we have

$$\begin{aligned} {{\,\mathrm{Tr}\,}}e^{-\beta H_\ell }&\le \sum _{n=0}^{\lfloor S\ell \rfloor } e^{ {-2\beta S}{\ell ^{-2}} n } {{\,\mathrm{Tr}\,}}\mathbb {1}_{T=S\ell -n} \\&= \sum _{n=0}^{\lfloor S\ell \rfloor } e^{ {-2\beta S}{\ell ^{-2}} n } \left( 2(S\ell -n)+1\right) {{\,\mathrm{Tr}\,}}\mathbb {1}_{T=S\ell -n}\mathbb {1}_{S_\mathrm{tot}^3 = n-S\ell } \\&\le (2 S\ell +1) \sum _{n=0}^{\lfloor S\ell \rfloor } e^{ {-2\beta S}{\ell ^{-2}} n } {{\,\mathrm{Tr}\,}}\mathbb {1}_{S_\mathrm{tot}^3 = n-S\ell }. \end{aligned}$$

The last trace equals the number of ways *n* indistinguishable particles can be distributed over $\ell$ sites, with at most 2*S* particles per site. Dropping this latter constraint for an upper bound, we obtain

$$\begin{aligned} {{\,\mathrm{Tr}\,}}e^{-\beta H_\ell } \le (2S \ell +1) \left( 1 - e^{ {-2\beta S}{\ell ^{-2}} }\right) ^{-\ell } \,. \end{aligned}$$

In particular,

5.6 $$\begin{aligned} f_\ell (\beta ,S) \ge -\frac{1}{\beta \ell } \ln (1+ 2 S\ell ) + \frac{1}{\beta } \ln \left( 1 - e^{ {-2\beta S}{\ell ^{-2}} }\right) \,. \end{aligned}$$

For large $\beta S$, this expression is minimized when $\ell \approx \ell _0$ with $\ell _0$ given in (5.4). If $\ell _0/2 \le \ell \le \ell _0$, we can use the lower bound on $\ell$ in the first term in (5.6), and the upper bound on the second, to obtain

5.7 $$\begin{aligned} f_\ell (\beta ,S) \ge -\frac{ (\ln \beta S)^{1/2}}{\beta ( \beta S)^{1/2}} \ln \left( 1+ 2 S (\beta S)^{1/2} (\ln \beta S)^{-1/2} \right) + \frac{1}{\beta } \ln \left( 1 - (\beta S)^{-1/2} \right) \,,\nonumber \\ \end{aligned}$$

which is of the desired form. If $\ell > \ell _0$, we can divide the interval $[1,\ell ]$ into smaller ones of size between $\ell _0/2$ and $\ell _0$. Using the subadditivity (5.1), we conclude (5.7) also in that case. $\square$

*Step 4. Restriction to low energies.* For any $E>0$, we have

$$\begin{aligned} {{\,\mathrm{Tr}\,}}e^{-\beta H_\ell }&\le {{\,\mathrm{Tr}\,}}e^{-\beta H_\ell } \mathbb {1}_{H_\ell< E} + e^{-\beta E/2} {{\,\mathrm{Tr}\,}}e^{-\beta H_\ell /2 } \mathbb {1}_{H_\ell \ge E} \\&\le {{\,\mathrm{Tr}\,}}e^{-\beta H_\ell } \mathbb {1}_{H_\ell < E} + e^{-\beta (E + \ell f_\ell (\beta /2,S))/2}. \end{aligned}$$

In particular, with the choice

$$\begin{aligned} E = E_0(\ell ,\beta ,S) := - \ell f_\ell (\beta /2,S) \end{aligned}$$

this gives

5.8 $$\begin{aligned} {{\,\mathrm{Tr}\,}}e^{-\beta H_\ell } \le 1 + {{\,\mathrm{Tr}\,}}e^{-\beta H_\ell } \mathbb {1}_{H_\ell < E_0}. \end{aligned}$$

Using the *SU*(2) invariance, we can further write

5.9 $$\begin{aligned} {{\,\mathrm{Tr}\,}}e^{-\beta H_\ell } \mathbb {1}_{H_\ell< E_0}&= \sum _{n=0}^{\lfloor S\ell \rfloor } (2(S\ell -n) +1) {{\,\mathrm{Tr}\,}}e^{-\beta H_\ell } \mathbb {1}_{H_\ell < E_0} \mathbb {1}_{T=S\ell -n} \mathbb {1}_{S_\mathrm{tot}^3=n-S\ell } \nonumber \\&\le (2 S\ell +1) \sum _{n=0}^{\lfloor S\ell \rfloor } {{\,\mathrm{Tr}\,}}e^{-\beta H_\ell } P_{E_0,n} \end{aligned}$$

where

5.10 $$\begin{aligned} P_{E_0,n} = \mathbb {1}_{H_\ell < E_0} \mathbb {1}_{T=S\ell -n} \mathbb {1}_{S_\mathrm{tot}^3=n-S\ell }. \end{aligned}$$

In other words, we can restrict the trace to states with $S_\mathrm{tot}^3$ being as small as possible (given $\vec {S}_\mathrm{tot}^2$). In the particle picture discussed in Sect. 3, this amounts to particle number ${\mathcal {N}} = S\ell - T = n$. Because of (5.3), we have $E_0 > H_\ell \ge 2 S n/\ell ^2$ on the range of $P_{E_0,n}$, hence the sum in (5.9) is restricted to

5.11 $$\begin{aligned} n < N_0 := \frac{E_0 \ell ^2}{2S}. \end{aligned}$$

*Step 5. A Laplacian lower bound.* With the aid of the Holstein–Primakoff representation (3.1), we can equivalently write the Hamiltonian $H_\ell$ in terms of bosonic creation and annihilation operators as

5.12 $$\begin{aligned} H_\ell = S\sum _{x=1}^{\ell -1} \left( a^\dagger _{x+1} \sqrt{ 1 - \frac{n_x}{2S} } - a^\dagger _x \sqrt{ 1 - \frac{n_{x+1}}{2S}} \right) \left( a_{x+1} \sqrt{ 1 - \frac{n_x}{2S} } - a_x \sqrt{ 1 - \frac{n_{x+1}}{2S}} \right) \nonumber \\ \end{aligned}$$

where $n_x =a^\dagger _x a_x \le 2S$. Note that written in this form, the Hamiltonian $H_\ell$ is manifestly positive, contrary to (3.2).

Let ${\mathcal {N}} = \sum _{x} n_x = \ell S + S_\mathrm{tot}^3$ denote the total number of bosons. States $\Psi$ with *n* particles, i.e., ${\mathcal {N}} \Psi = n\Psi$, are naturally identified with *n*-boson wave functions^1^ in $\ell ^2_\mathrm{sym}([1,\ell ]^n)$ via

$$\begin{aligned} \Psi = \frac{1}{\sqrt{n!}} \sum _{1\le x_1,\ldots ,x_n \le \ell } \Psi (x_1,\ldots ,x_n) { a^\dagger _{x_1} \ldots a^\dagger _{x_n} |\Omega \rangle } \,, \end{aligned}$$

where $|\Omega \rangle$ denotes the vacuum (which corresponds to the state with all spins pointing maximally down). Using (5.12), we have in this representation

$$\begin{aligned} \langle \Psi | H_\ell \Psi \rangle&= S n \sum _{x=1}^{\ell -1} \sum _{x_1,\ldots ,x_{n-1}} \left| \Psi (x+1,x_1,\ldots ,x_{n-1}) \sqrt{ 1 - \frac{\sum _{k=1}^{n-1} \delta _{x,x_k}}{2S} } \right. \\&\left. \qquad \qquad \qquad \qquad \qquad - \Psi (x,x_1,\ldots ,x_{n-1}) \sqrt{ 1 - \frac{\sum _{k=1}^{n-1} \delta _{x+1,x_k}}{2S} }\right| ^2. \end{aligned}$$

Because of permutation-symmetry, we can also write this as

$$\begin{aligned} \langle \Psi | H_\ell \Psi \rangle&= S \sum _{j=1}^n \sum _{\begin{array}{c} x_1,\ldots ,x_{n} \\ x_j \le \ell -1 \end{array}} \left| \Psi (x_1,\ldots , x_j+1, \ldots x_{n}) \sqrt{ 1 - \frac{\sum _{k, k\ne j} \delta _{x_j,x_k}}{2S} } \right. \\&\left. \qquad \qquad \qquad \qquad \quad - \Psi (x_1,\ldots ,x_j, \ldots x_{n}) \sqrt{ 1 - \frac{\sum _{k, k\ne j} \delta _{x_j+1,x_k}}{2S} }\right| ^2\,. \end{aligned}$$

For a lower bound, we can restrict the sum over $x_1,\ldots ,x_{n}$ to values such that $x_k\ne x_l$ for all $k\ne \ell$. For a given *j*, we can further restrict to $x_k\ne x_j+1$ for all $k\ne j$. In this case, the square root factors above are equal to 1. In other words, we have the lower bound

$$\begin{aligned} \langle \Psi | H_\ell \Psi \rangle \ge \frac{S}{2} \sum _{\begin{array}{c} X,Y \in {\mathcal {X}}_{\ell ,n} \\ |X-Y| = 1 \end{array}} \left| \Psi (X) - \Psi (Y)\right| ^2 \end{aligned}$$

where the sum is over the set ${\mathcal {X}}_{\ell ,n} := \{ [1,\ell ]^n : x_i \ne x_j \forall i\ne j\}$, and $|X-Y| = \sum _{i=1}^n |x_i-y_i|$. Note that we have to assume that $\ell \ge n$ for the set ${\mathcal {X}}_{\ell ,n}$ to be non-empty. The factor 1/2 arises from the fact that particles are allowed to hop both left and right, i.e., each pair (*X*, *Y*) appears twice in the sum. Note also that the above inequality is actually an equality for $S=1/2$, since in this case no two particles can occupy the same site.

On the set $\{ 1\le x_1< x_2< \cdots <x_n\le \ell \} \subset {\mathcal {X}}_{\ell ,n}$, define the map

$$\begin{aligned} V (x_1, \ldots , x_n) = (x_1, x_2-1, x_3-2 , \ldots , x_n - n+ 1) \end{aligned}$$

and extend it to the set ${\mathcal {X}}_{\ell ,n} = \{ [1,\ell ]^n : x_i \ne x_j \forall i\ne j\}$ via permutations. In other words, *V* maps $x_i$ to $x_i - k_i$ where $k_i$ denotes the number of $x_j$ with $x_j < x_i$. As a map from ${\mathcal {X}}_{\ell ,n}$ to $[1,\ell -n+1]^n$, *V* is clearly surjective, but it is not injective. Points in $[1,\ell -n+1]^n$ with at least two coordinates equal have more than one pre-image under *V*. The pre-images are unique up to permutations, however, hence we can define a map ${\mathbb {V}}: \ell ^2_\mathrm{sym}([1,\ell ]^n) \rightarrow \ell ^2_\mathrm{sym}([1,\ell -n+1]^n)$ via

5.13 $$\begin{aligned} {\mathbb {V}} \Psi (V(X)) = \Psi (X) \quad \text {for } X\in {\mathcal {X}}_{\ell ,n}. \end{aligned}$$

We then have

$$\begin{aligned}&\sum _{\begin{array}{c} X,Y \in {\mathcal {X}}_{\ell ,n} \\ |X-Y| = 1 \end{array}} \left| \Psi (X) - \Psi (Y)\right| ^2 \\&\quad = \sum _{A,B \in [1,\ell -n+1]^n} \left| {\mathbb {V}} \Psi (A)- {\mathbb {V}} \Psi (B) \right| ^2 \sum _{X\in V^{-1}(A), Y\in V^{-1}(B)} \chi _{|X-Y|=1}\,. \end{aligned}$$

For every pair $(A,B)\in [1,\ell -n+1]^n$ with $|A-B|=1$, there exists at least one pair $(X,Y)\in {\mathcal {X}}_{\ell ,n}$ with $|X-Y|=1$ in the pre-image of *V*. In other words, the last sum above is greater or equal to 1 if $|A-B|=1$. We have thus proved the following statement.

### Proposition 5.2

Let ${\mathbb {V}}: \ell ^2_\mathrm{sym}([1,\ell ]^n) \rightarrow \ell ^2_\mathrm{sym}([1,\ell -n+1]^n)$ be defined in (5.13). Then,

$$\begin{aligned} \mathbb {1}_{{\mathcal {N}}=n} H_\ell \ge S {\mathbb {V}}^\dagger (-\Delta _n^{\ell -n+1}) {\mathbb {V}}, \end{aligned}$$

where $\Delta _n^\ell$ denotes the Laplacian^2^ on $[1,\ell ]^n$.

*Step 6. Bounds on the two-particle density.* We will use Proposition 5.2 and the min–max principle to obtain a lower bound on the eigenvalues of $H_\ell$. For this purpose, we need an estimate on the norm of ${\mathbb {V}}\Psi$.

For $\Psi \in \ell ^2_\mathrm{sym}([1,\ell ]^n)$ with $\Vert \Psi \Vert =1$, we let

$$\begin{aligned} \rho _\Psi (x,y) = \langle \Psi | a^\dagger _x a^\dagger _y a_y a_x \Psi \rangle \end{aligned}$$

denote its two-particle density.

### Lemma 5.3

Let $\Psi \in \ell ^2_\mathrm{sym}([1,\ell ]^n)$ with $\Vert \Psi \Vert =1$. Then,

5.14 $$\begin{aligned} \Vert {\mathbb {V}}\Psi \Vert ^2 \ge 1 - \frac{1}{2} \sum _{x=1}^\ell \rho _\Psi (x,x) - \sum _{x=1}^{\ell -1} \rho _\Psi (x,x+1)\,. \end{aligned}$$

### Proof

From the definition of $\Phi :={\mathbb {V}}\Psi$, we have

$$\begin{aligned} \Vert \Phi \Vert ^2 = \sum _{A\in [1,\ell -n+1]^n} | \Phi (A)|^2 = \sum _{X\in {\mathcal {X}}_{\ell ,n}} |\Psi (X)|^2 | V^{-1}(V(X))|^{-1} \,, \end{aligned}$$

where $|V^{-1}(V(X))|$ denotes the number of points in the pre-image of *V*(*X*). This number equals one if *X* is such that $|x_j-x_k|\ge 2$ for all $j\ne k$. Hence,

$$\begin{aligned} \Vert \Phi \Vert ^2 \ge \sum _{\begin{array}{c} X\in {\mathcal {X}}_{\ell ,n} \\ |x_j - x_k|\ge 2 \, \forall j\ne k \end{array}} |\Psi (X)|^2\ge & {} \Vert \Psi \Vert ^2 - \frac{1}{2} \sum _{x=1}^\ell \langle \Psi | n_x(n_x-1) \Psi \rangle \\&- \sum _{x=1}^{\ell -1} \langle \Psi | n_x n_{x+1} \Psi \rangle . \end{aligned}$$

Indeed, the norm of $\Psi$ involves a sum over all possible configurations so we need to remove the terms which correspond to $x_i=x_j$ or $x_i=x_j+1$ for some $i \ne j$. The $x_i=x_j$ terms are removed through the term $\frac{1}{2} \sum _{x=1}^\ell n_x(n_x-1)$, which is zero if and only if on each site there is at most one particle. Similarly, the terms corresponding to $x_i=x_j+1$ are removed through $\sum _{x=1}^{\ell -1} n_x n_{x+1}$, which is zero if and only if there are no two neighboring sites that are occupied. With $\Vert \Psi \Vert =1$ and the definition of $\rho _\Psi (x,y)$ this becomes (5.14). $\square$

We shall give a lower bound on the right side of (5.14) in terms of the energy of $\Psi$.

### Proposition 5.3

Let $\Psi \in \ell ^2_\mathrm{sym}([1,\ell ]^n)$ with $\Vert \Psi \Vert =1$. Then,

5.15 $$\begin{aligned} \sum _{x=1}^{\ell -1} \rho _\Psi (x+1,x) \le \frac{4}{\ell }n(n-1) + 4 (n-1) \sqrt{\frac{n}{S}} \langle \Psi | H_\ell \Psi \rangle ^{1/2}. \end{aligned}$$

### Proof

For $x\ne z$, we have

$$\begin{aligned}&\rho _\Psi (x,y)\left( 1- \frac{\delta _{z,y}}{2S}\right) - \rho _\Psi (z,y) \left( 1 -\frac{\delta _{x,y}}{2S}\right) \\&\quad = \mathfrak {R}\left\langle \Psi \left| \left( a^\dagger _x\sqrt{1-\frac{n_z}{2S}} - a^\dagger _z \sqrt{1-\frac{n_x}{2S}} \right) n_y \left( a_x\sqrt{1-\frac{n_z}{2S}} + a_z \sqrt{1-\frac{n_x}{2S}} \right) \right. \Psi \right\rangle \,. \end{aligned}$$

The Cauchy–Schwarz inequality therefore implies that

$$\begin{aligned}&\left| \rho _\Psi (x,y)\left( 1- \frac{\delta _{z,y}}{2S}\right) - \rho _\Psi (z,y) \left( 1 -\frac{\delta _{x,y}}{2S}\right) \right| ^2\\&\quad \le \left\langle \Psi \left| \left( a^\dagger _x\sqrt{1-\frac{n_z}{2S}} - a^\dagger _z \sqrt{1-\frac{n_x}{2S}} \right) n_y \left( a_x\sqrt{1-\frac{n_z}{2S}} - a_z \sqrt{1-\frac{n_x}{2S}} \right) \right. \Psi \right\rangle \\&\qquad \times \left\langle \Psi \left| \left( a^\dagger _x\sqrt{1-\frac{n_z}{2S}} + a^\dagger _z \sqrt{1-\frac{n_x}{2S}} \right) n_y \left( a_x\sqrt{1-\frac{n_z}{2S}} + a_z \sqrt{1-\frac{n_x}{2S}} \right) \right. \Psi \right\rangle \,. \end{aligned}$$

Moreover,

$$\begin{aligned}&\left\langle \Psi \left| \left( a^\dagger _x\sqrt{1-\frac{n_z}{2S}} + a^\dagger _z \sqrt{1-\frac{n_x}{2S}} \right) n_y \left( a_x\sqrt{1-\frac{n_z}{2S}} + a_z \sqrt{1-\frac{n_x}{2S}} \right) \right. \Psi \right\rangle \\&\quad \le 2 \left\langle \Psi \left| a^\dagger _x \left( 1-\frac{n_z}{2S}\right) n_y a_x \right. \Psi \right\rangle + 2 \left\langle \Psi \left| a^\dagger _z \left( 1-\frac{n_x}{2S}\right) n_y a_z \right. \Psi \right\rangle \\&\quad \le 2 \rho _\Psi (x,y) \left( 1-\frac{\delta _{z,y}}{2S}\right) + 2 \rho _\Psi (z,y) \left( 1-\frac{\delta _{x,y}}{2S}\right) \,. \end{aligned}$$

With

$$\begin{aligned} h_x^y := \left( a^\dagger _{x+1}\sqrt{1-\frac{n_x}{2S}} - a^\dagger _x \sqrt{1-\frac{n_{x+1}}{2S}} \right) n_y \left( a_{x+1}\sqrt{1-\frac{n_x}{2S}} - a_x \sqrt{1-\frac{n_{x+1}}{2S}} \right) , \end{aligned}$$

we thus have

5.16 $$\begin{aligned}&\left| \rho _\Psi (x+1,y)\left( 1- \frac{\delta _{x,y}}{2S}\right) - \rho _\Psi (x,y) \left( 1 -\frac{\delta _{x+1,y}}{2S}\right) \right| ^2 \nonumber \\&\quad \le 2 \left\langle \Psi \left| h_x^y \right. \Psi \right\rangle \left( \rho _\Psi (x+1,y) \left( 1-\frac{\delta _{x,y}}{2S}\right) + \rho _\Psi (x,y) \left( 1-\frac{\delta _{x+1,y}}{2S}\right) \right) \,. \end{aligned}$$

We note that

$$\begin{aligned} S\sum _{x=1}^{\ell -1} \sum _{y=1}^\ell h_x^y = H_\ell \left( {\mathcal {N}} -1\right) \,. \end{aligned}$$

For given $y \le \ell /2$, choose $x_y > y$ such that

$$\begin{aligned} \rho _\Psi (x,y) \ge \rho _\Psi (x_y, y) \quad \text{ for } \text{ all } x > y\,. \end{aligned}$$

We have

$$\begin{aligned} \rho _\Psi (y+1,y) = \rho _\Psi (x_y,y) + \sum _{w = y+1}^{x_y-1} \left( \rho _\Psi (w,y) - \rho _\Psi (w+1,y) \right) \end{aligned}$$

(where the sum is understood to be zero if $x_y=y+1$). The first term on the right side can be bounded as

$$\begin{aligned} \rho _\Psi (x_y,y) \le \frac{1}{\ell -y} \sum _{x=y+1}^\ell \rho _\Psi (x,y) \le \frac{2}{\ell }\sum _{x=1}^\ell \rho _\Psi (x,y) \end{aligned}$$

using that $y\le \ell /2$ by assumption. For the second, we use the bound (5.16) above, which implies that

$$\begin{aligned} \left| \rho _\Psi (w,y) - \rho _\Psi (w+1,y) \right| \le \sqrt{2} \langle \Psi | h_w^y \Psi \rangle ^{1/2} \left( \rho _\Psi (w+1,y) + \rho _\Psi (w,y) \right) ^{1/2} \end{aligned}$$

for $w\ge y+1$. After summing over *y* and *w*, using the Cauchy–Schwarz inequality and the fact that $\sum _{x,y}\rho _\Psi (x,y) = n(n-1)$, we thus have the upper bound

$$\begin{aligned} \sum _{y\le \ell /2} \rho _\Psi (y+1,y) \le \frac{2n(n-1)}{\ell }+ 2 \sqrt{\frac{n}{S}}(n-1) \langle \Psi | H_\ell \Psi \rangle ^{1/2}. \end{aligned}$$

If $y>\ell /2$, we use the symmetry of $\rho$ and write

$$\begin{aligned} \rho _\Psi (y+1,y) = \rho _\Psi (y,y+1)= & {} \rho _\Psi (x_y,y+1) \\&+ \sum _{w=x_y}^{y-1} \left( \rho _\Psi (w+1 ,y+1) - \rho _\Psi (w, y+1) \right) \end{aligned}$$

instead, where $x_y$ is now defined by minimizing $\rho _\Psi (x,y+1)$ for $x\le y$. Proceeding as above, we finally conclude the desired estimate. $\square$

A similar bound holds for $\sum _x \rho _\Psi (x,x)$.

### Proposition 5.4

Let $\Psi \in \ell ^2_\mathrm{sym}([1,\ell ]^n)$ with $\Vert \Psi \Vert =1$. Then,

5.17 $$\begin{aligned} \sum _{x=1}^\ell \rho _\Psi (x,x) \le \frac{4}{\ell }n(n-1) + (4 + \sqrt{3}) (n-1) \sqrt{\frac{n}{S}} \langle \Psi | H_\ell \Psi \rangle ^{1/2} \,. \end{aligned}$$

### Proof

Since $\rho _\Psi (x,x)$ vanishes for $S=1/2$, we can assume $S\ge 1$ henceforth. By (5.16),

$$\begin{aligned}&\left| \rho _\Psi (x\pm 1,x)\left( 1- \frac{1}{2S}\right) - \rho _\Psi (x,x) \right| ^2\\&\quad \le 2 \sum _{y=1}^{\ell -1} \left\langle \Psi \left| h_y^x \right. \Psi \right\rangle \left( \rho _\Psi (x\pm 1,x) \left( 1-\frac{1}{2S}\right) + \rho _\Psi (x,x) \right) \,. \end{aligned}$$

It thus follows from the Cauchy–Schwarz inequality that

$$\begin{aligned}&\sum _{x=1}^\ell \rho _\Psi (x,x) \le 2 \left( 1 -\frac{1}{2S}\right) \sum _{x=1}^{\ell -1} \rho _\Psi (x+1,x) \\&\quad + \sqrt{2(n-1) /S } \left\langle \Psi | H_\ell \Psi \right\rangle ^{1/2} \left( 2 \sum _{x=1}^{\ell -1} \rho _\Psi (x+ 1,x) \left( 1-\frac{1}{2S}\right) + \sum _{x=1}^\ell \rho _\Psi (x,x) \right) ^{1/2}\,. \end{aligned}$$

In the last line, we can make the rough bounds $2 \sum _{x=1}^{\ell -1} \rho _\Psi (x+ 1,x) \le n(n-1)$ and $\sum _{x=1}^\ell \rho _\Psi (x,x)\le n(n-1)$, and for the term in the first line we use (5.15). Using also $S\ge 1$, this completes the proof of (5.17). $\square$

*Step 7. Final estimate.* Recall the definition (5.10) of $P_{E_0,n}$. It follows from Proposition 5.2 that

$$\begin{aligned} P_{E_0,n} H_\ell \ge S P_{E_0,n} {\mathbb {V}}^\dagger (-\Delta _n^{\ell -n+1}) {\mathbb {V}}P_{E_0,n} \end{aligned}$$

and from Lemma 5.3 and Propositions 5.3 and 5.4 that

$$\begin{aligned} P_{E_0,n} {\mathbb {V}}^\dagger {\mathbb {V}} P_{E_0,n} \ge P_{E_0,n} (1 - \delta ) \end{aligned}$$

where

5.18 $$\begin{aligned} \delta = \frac{8N_0^2}{\ell }+ 9 N_0 \sqrt{\frac{N_0 E_0}{S}} = \left( 2 + \frac{9}{\sqrt{8}} \right) \frac{E_0^2 \ell ^3}{S^2}. \end{aligned}$$

Here, we used (5.11). We shall choose the parameters such that $\delta \ll 1$ for large $\beta$. The min–max principle readily implies that the eigenvalues of $H_\ell$ in the range $P_{E_0,n}$ are bounded from below by the corresponding ones of $S(1-\delta )(-\Delta _n^{\ell -n+1})$. In particular, for any $\beta >0$

$$\begin{aligned} {{\,\mathrm{Tr}\,}}P_{E_0,n} e^{-\beta H_\ell } \le {{\,\mathrm{Tr}\,}}e^{\beta S(1-\delta ) \Delta _n^{\ell -n+1}}\,. \end{aligned}$$

Note that the Laplacian $\Delta _n^{\ell -n+1}$ depends on *n*, besides the particle number, also via the size of the interval $[1,\ell -n+1]$. For a lower bound, we can increase the interval size back to $\ell$, all eigenvalues are clearly decreasing under this transformation. In particular,

5.19 $$\begin{aligned} {{\,\mathrm{Tr}\,}}e^{-\beta H_\ell } \mathbb {1}_{H_\ell < E_0}&\le (2 S\ell +1) \sum _{n=0}^{\lfloor N_0\rfloor } {{\,\mathrm{Tr}\,}}e^{\beta S(1-\delta ) \Delta _n^{\ell }} \nonumber \\&\le (2 S\ell +1) (N_0+1 ) \prod _{m=1}^{\ell -1} \left( 1 - e^{-\beta S (1-\delta ) \varepsilon (\pi m/\ell ) } \right) ^{-1} \end{aligned}$$

where $\varepsilon (p) = 2 (1-\cos p)$ is the dispersion relation of the discrete Laplacian on $[1,\ell ]$.

Combining (5.8) and (5.19), we have thus shown that

$$\begin{aligned} f_\ell (\beta ,S)&\ge -\frac{1}{\beta \ell } \ln \left( 1+ (2S\ell +1) (N_0+1 ) \prod _{m=1}^{\ell -1} \left( 1 - e^{-\beta S (1-\delta ) \varepsilon (\pi m/\ell ) } \right) ^{-1}\right) \\&\ge \frac{1}{\beta \ell } \sum _{m=1}^{\ell -1} \ln \left( 1 - e^{-\beta S (1-\delta ) \varepsilon (\pi m/\ell ) } \right) -\frac{1}{\beta \ell } \ln \left( 1+ (2S\ell +1) (N_0+1 ) \right) \,, \end{aligned}$$

with $\delta$ in (5.18), $N_0 = E_0 \ell ^2/(2S)$ and $E_0 = {\mathcal {O}}( \ell \beta ^{-3/2} S^{-1/2} ( \ln (\beta S))^{1/2} \ln (\beta S^3))$. Since $\varepsilon (p)$ is increasing in *p*, we further have

$$\begin{aligned} \frac{1}{\beta \ell } \sum _{m=1}^{\ell -1} \ln \left( 1 - e^{-\beta S (1-\delta ) \varepsilon (\pi m/\ell ) } \right) \ge \frac{1}{\pi \beta } \int _0^\pi \ln (1-e^{-\beta S (1-\delta ) \varepsilon (p)}) \mathrm{d}p. \end{aligned}$$

The error terms compared to the desired expression

$$\begin{aligned} \frac{1}{\pi \beta } \int _0^\pi \ln (1-e^{-\beta S \varepsilon (p)}) \mathrm{d}p = {\mathcal {O}}\left( \beta ^{-3/2} S^{-1/2}\right) \end{aligned}$$

are thus

$$\begin{aligned} \ell ^5 \frac{\ln (\beta S)}{ (\beta S)^3} \left( \ln (\beta S^3) \right) ^2 \quad \text {and} \quad (\beta S)^{1/2}\ell ^{-1} \ln \left( S \ell N_0\right) \end{aligned}$$

which leads to a choice of $\ell = C (\beta S)^{1/2 + 1/12} (\ln (\beta S^3))^{-1/3}$ and a relative error of the order $(\beta S)^{-1/12} \ln (\beta S) (\ln (\beta S^3))^{1/3}$. Note that for this

choice the condition $\ell \ge \ell _0/2$ of Lemma 5.2 is fulfilled exactly when this error is small.

Finally, we note that (compare with [13, Eqs. (5.42) and (5.43)])

$$\begin{aligned} \int _0^\pi \ln (1-e^{-\beta S \varepsilon (p)}) \mathrm{d}p \ge \frac{1}{ (\beta S)^{1/2}} \int _0^\infty \ln (1-e^{-p^2}) \mathrm{d}p - {\mathcal {O}}( (\beta S)^{-3/2}) \end{aligned}$$

for large $\beta S$. This completes the proof of the lower bound. $\square$

## Appendix A: Upper bound in two dimensions

In two dimensions, we consider the ferromagnetic Heisenberg model with nearest neighbor interactions on the square lattice ${\mathbb {Z}}^2$. It is defined in terms of the Hamiltonian

A.1 $$\begin{aligned} H_\Lambda := \sum _{\langle x,y \rangle \subset \Lambda }(S^2- {\vec {S}}_{x} \cdot {\vec {S}}_y)\,, \end{aligned}$$

where $\langle x,y \rangle$ denotes a pair of nearest neighbors and $\Lambda$ is a finite subset of ${\mathbb {Z}}^2$. We denote the free energy in the thermodynamic limit by

A.2 $$\begin{aligned} f^\mathrm{{2d}}(\beta ,S) : = \lim _{\Lambda \rightarrow {\mathbb {Z}}^2} f^\mathrm{2d}_{\Lambda }(\beta ,S) = - \lim _{\Lambda \rightarrow {\mathbb {Z}}^2} \frac{1}{\beta |\Lambda |} {{\,\mathrm{Tr}\,}}e^{-\beta H_\Lambda } \,. \end{aligned}$$

The limit has to be understood via a suitable sequence of increasing domains, e.g., squares of side length *L* with $L\rightarrow \infty$.

For $d=2$, we have the following upper bound.

### Proposition A.1

Consider the Hamiltonian (A.1) and the corresponding free energy (A.2). Let

A.3 $$\begin{aligned} C_2:=\frac{1}{(2\pi )^2}\int _{{\mathbb {R}}^2}\ln \big (1-e^{-p^2}\big )\mathrm{d}p = \frac{-\zeta (2)}{4\pi }=-\frac{\pi }{24}. \end{aligned}$$

Then, for any $S\ge 1/2$, we have

A.4 $$\begin{aligned} f^\mathrm{{2d}}(\beta ,S) \le C_2 S^{-1} \beta ^{-2} \left( 1 - {\mathcal {O}}( (\beta S)^{-1/3} ( \ln \beta S)^{2/3}) \right) \end{aligned}$$

as $$\beta S \rightarrow \infty $$.

We note that it remains an open problem to derive a corresponding lower bound, i.e., the analogue of Proposition 5.1 in $d=2$ dimensions.

The proof of Proposition A.1 differs from the one-dimensional case discussed in Sect. 4 only in the evaluation of the error terms in Lemmas 4.2 and 4.3. Let $\Gamma$, ${\mathcal {P}}$ and *K* be defined as in (4.4), (4.5) and (4.7), with the obvious modifications to $d=2$, for a square-shaped domain $\Lambda _\ell = [1,\ell ]^2$. Then, the following holds

### Lemma A.1

In the case $d=2$, we have

A.5 $$\begin{aligned} \frac{ {{\,\mathrm{Tr}\,}}_{\mathcal {F}}{\mathcal {P}} e^{-\beta K}{\mathcal {P}}}{{{\,\mathrm{Tr}\,}}_{\mathcal {F}}e^{-\beta K} } \ge 1- \left( \frac{\pi \ell \ln (1+2 \ell )}{2\beta S}\right) ^2 \,. \end{aligned}$$

### Proof

The bound (4.15) remains correct in two dimensions. We thus only need to estimate the (now) double sum over the two-dimensional dual lattice

$$\begin{aligned} \frac{ {{\,\mathrm{Tr}\,}}_{\mathcal {F}}n_x e^{-\beta K}}{{{\,\mathrm{Tr}\,}}_{\mathcal {F}}e^{-\beta K}}\le \sum _{p \in \Lambda _\ell ^{*\mathrm D}}\frac{|\phi _p(x)|^2}{e^{\beta S \varepsilon (p)}-1}\le \frac{4}{(\ell +1)^2}\sum _{m=1}^\ell \sum _{n=1}^\ell \frac{1}{e^{\beta S {\tilde{\varepsilon }} (m, n)}-1} \end{aligned}$$

where ${\tilde{\varepsilon }} (m,n)=2(2-\cos (\frac{\pi m}{\ell +1})-\cos (\frac{\pi n}{\ell +1}))$. By proceeding as in the proof of Lemma 4.2, we have

A.6 $$\begin{aligned} \frac{ {{\,\mathrm{Tr}\,}}_{\mathcal {F}}n_x e^{-\beta K}}{{{\,\mathrm{Tr}\,}}_{\mathcal {F}}e^{-\beta K}}\le \frac{1}{\beta S} \sum _{m=1}^\ell \sum _{n=1}^\ell \frac{1}{m^2 + n^2} \le \frac{\pi }{2} \frac{ \ln (1+ 2\ell )}{\beta S} \,. \end{aligned}$$

Looking again at (4.15), we see that the summation over $x\in \Lambda _\ell$ yields a factor $\ell ^2$, and hence we arrive at the desired bound (A.5). $\square$

Next, we establish the two-dimensional counterpart of the entropy estimate. We have

### Lemma A.2

In the case $d=2$, we have

$$\begin{aligned} \frac{1}{\beta }{{\,\mathrm{Tr}\,}}\Gamma \ln \Gamma&\le - \frac{1}{\beta }\ln \left( {{\,\mathrm{Tr}\,}}_{\mathcal {F}}{\mathcal {P}} e^{-\beta K}{\mathcal {P}}\right) - \frac{{{\,\mathrm{Tr}\,}}_{\mathcal {F}}K e^{-\beta K} }{{{\,\mathrm{Tr}\,}}_{\mathcal {F}}{\mathcal {P}} e^{-\beta K}{\mathcal {P}}} \\&\quad + \frac{S}{2} \left( \frac{\pi }{2} \ell (\ell +1) \frac{\ln (1+2\ell )}{(\beta S)^2} \right) ^2 \left[ \frac{\pi ^3}{48} + \frac{\beta S}{\ell ^2} \right] \frac{{{\,\mathrm{Tr}\,}}_{\mathcal {F}}e^{-\beta K} }{{{\,\mathrm{Tr}\,}}_{\mathcal {F}}{\mathcal {P}} e^{-\beta K}{\mathcal {P}}} \,. \end{aligned}$$

### Proof

As in the case of the previous lemma, the only difference with regard to the one-dimensional case lies in the estimation of the *p* sums in (4.18). By proceeding similarly as above, we obtain

$$\begin{aligned} \sum _{p \in \Lambda _\ell ^{*\mathrm D}} \frac{2S \varepsilon (p) }{e^{\beta S \varepsilon (p)} -1} \le \frac{\pi ^3}{48} S \frac{(\ell +1)^2}{(\beta S)^{2}} \end{aligned}$$

as well as

$$\begin{aligned} \sum _{p \in \Lambda _\ell ^{*\mathrm D}} \frac{ S\varepsilon (p) }{ \left( \sinh \tfrac{1}{2} \beta S \varepsilon (p) \right) ^2 } \le S \frac{ (\ell +1)^2}{(\beta S)^2} \sum _{m=1}^\ell \sum _{n=1}^\ell \frac{1}{m^2 + n^2} \le \frac{\pi }{2} S \frac{ (\ell +1)^2}{(\beta S)^2} \ln (1+2\ell )\,. \end{aligned}$$

In combination with (A.6), this yields the desired result. $\square$

It remains to obtain the two-dimensional counterpart of the final estimate of the free energy. The Gibbs variational principle together with Lemma A.1 and Lemma A.2 implies that for $C (\beta S)^{1/2} \le \ell \ll \beta S / \ln (\beta S)$

$$\begin{aligned} \begin{aligned} f_{\Lambda _\ell }^{2d,\mathrm D}(\beta ,S)&\le -\frac{1}{\beta \ell ^2}\ln \left( {{\,\mathrm{Tr}\,}}_{\mathcal {F}}e^{-\beta K} \right) -\frac{1}{\beta \ell ^2}\ln \left( 1-\frac{C \ell ^2 \ln ^2 \ell }{(\beta S)^2}\right) + CS\frac{\ell ^2 \ln ^2 \ell }{(\beta S)^4} \end{aligned} \end{aligned}$$

for a suitable constant $C>0$. The first term on the right side equals

A.7 $$\begin{aligned} - \frac{1}{\beta \ell ^2} \ln \left( {{\,\mathrm{Tr}\,}}_{\mathcal {F}}e^{-\beta K} \right) = \frac{1}{\beta \ell ^2} \sum _{p\in \Lambda _\ell ^{*\mathrm D} } \ln ( 1- e^{-\beta S \varepsilon (p)} )\,. \end{aligned}$$

By monotonicity, we can again bound the sum in terms of the corresponding integral, i.e.,

A.8 $$\begin{aligned} \frac{1}{\beta \ell ^2} \sum _{p\in \Lambda _\ell ^{*\mathrm D} } \ln ( 1- e^{-\beta S \varepsilon (p)} ) \le \frac{1}{\beta \pi ^2} \left( 1 + \ell ^{-1} \right) ^2 \int _{[\frac{\pi }{\ell +1},\pi ]^2} \ln ( 1- e^{-\beta S \varepsilon (p)} ) \mathrm{d}p \,.\nonumber \\ \end{aligned}$$

The missing term is now bounded by

$$\begin{aligned} - \frac{2}{\beta \pi ^2} \int _{[0,\frac{\pi }{\ell +1}]\times [0,\pi ]} \ln ( 1- e^{-\beta S \varepsilon (p)} ) \mathrm{d}p \le - \frac{1}{\beta (\beta S)^{1/2} (\ell +1)} \int _{{\mathbb {R}}_+} \ln ( 1- e^{-p^2 } ) \mathrm{d}p\,. \end{aligned}$$

Furthermore, since $\varepsilon (p)\le |p|^2$ we have

A.9 $$\begin{aligned} \frac{1}{ \pi ^2} \int _{[0,\pi ]^2} \ln ( 1- e^{-\beta S \varepsilon (p)} ) \mathrm{d}p&\le \frac{1}{ (2\pi )^2} \int _{{\mathbb {R}}^2} \ln ( 1- e^{-\beta S |p|^2} )\mathrm{d}p+ \frac{C}{ (\beta S)^\alpha } \nonumber \\&=C_2 (\beta S)^{-1} + \frac{C}{ (\beta S)^\alpha } \end{aligned}$$

for $\alpha >0$ arbitrary, some $C>0$ (depending on $\alpha$), and $C_2$ defined in (A.3). For $\ell$ satisfying $\ell \ln \ell \ll \beta S$ and $\ell \gg (\beta S)^{1/2}$, all the error terms are small compared to the main term. The desired upper bound stated in Proposition A.1 is obtained by choosing $\ell = C (\beta S)^{5/6} (\ln \beta S)^{-2/3}$. $\square$

## Footnotes

1 Here, $\ell ^2_\mathrm{sym}(A)$ denotes the Hilbert space of square-summable sequences on *A* invariant under permutations

2 This is the graph Laplacian, with free (or Neumann) boundary conditions.
